# Perioperative Management of Acquired Hemophilia A: A Case Report and Review of Literature

**DOI:** 10.5812/aapm.11906

**Published:** 2013-12-27

**Authors:** Yashar Ilkhchoui, Eugene Koshkin, Jimmy J Windsor, Timothy R Petersen, Matthew Charles, Jeffery D Pack

**Affiliations:** 1 Department of Anesthesiology and Critical Care Medicine, School of Medicine, University of New Mexico, Albuquerque, USA

**Keywords:** Factor Eight Inhibitor Bypassing Activity, Immunosuppressive Agents, Perioperative Care

## Abstract

**Introduction:**

Acquired hemophilia A is a rare bleeding disorder with a high mortality rate. Diagnosis and treatment of this disorder can be very challenging to anesthesiologists because of lack of a personal or familial abnormal bleeding history.

**Case presentation:**

We report a 60-year-old woman who presented to the operating room for an urgent fasciotomy. She was initially diagnosed to have compartment syndrome of her left upper extremity secondary to an expanding hematoma after multiple unsuccessful venipuncture attempts. After surgical intervention, she developed recurrent intramuscular hematomas, became severely anemic, and required surgical re-exploration and multiple blood product transfusions. Ultimately, she was found to have an elevated activated partial thromboplastin time (aPTT), very low FVIII activity, and high FVIII inhibitor titers consistent with the diagnosis of acquired hemophilia A.

**Conclusions:**

Treatment strategies in acquired hemophilia are based on two major objectives. During the acute stage, effective control of bleeding is critical. The ultimate therapeutic goal during the subacute phase is the elimination of the inhibitors targeting factor VIII. Here, we present this case and will review current literature regarding therapeutic approaches to this rare condition in the operating room setting and postoperative course.

## 1. Introduction

Acquired hemophilia A (AHA) is a rare, life-threatening bleeding disorder caused by autoantibodies against a coagulation factor, usually factor VIII (FVIII), and associated with significant morbidity and mortality (1). The diagnosis of AHA can be very challenging, as the affected patients do not have a familial or personal history of excessive bleeding (2). Appropriate perioperative management of these patients requires an in-depth understanding of AHA pathophysiology by anesthesiologists since the therapeutic options are different than for other bleeding disorders, mandating familiarity with the available medications and blood products used in treatment of this particular disorder.

## 2. Case Presentation

A 60-year-old woman with a history of rheumatoid arthritis, hypothyroidism, and asthma presented to the emergency department (ED) complaining of severe pain and swelling in her left upper extremity starting a day prior. She had visited the ED two days earlier with complaints of abdominal pain. At the first ED visit, intravenous (IV) access was unsuccessfully attempted multiple times in her left upper extremity to draw blood for laboratory tests and IV fluid administration. IV access was established in her other arm without difficulty, and she was discharged home after the initial work-up revealed no abnormal findings. However, during the second emergency department visit (two days after first visit), she was diagnosed with an expanding hematoma in her left arm, impending compartment syndrome, and a possible arterial injury. Therefore, surgical exploration, fasciotomy, and evacuation of a large hematoma were performed. She did not have any arterial injury. In the postoperative period she continued to have significant bleeding from her surgical wound; and her hemoglobin dropped from 12 g/dl to 5.2 g/dl. Her aPTT was elevated and platelet count was within normal limits. Another urgent surgical exploration under general anesthesia was warranted. During the second surgery, another large hematoma was evacuated and there was persistent blood oozing from the brachioradialis muscle, which did not stop with coagulation, direct pressure, epinephrine-soaked gauzes, thrombin spray, or hemostatic dressings. Four units of packed red blood cells (PRBC) and four units of fresh frozen plasma (FFP) were transfused until the bleeding was controlled. Hematology consult was obtained immediately, and extensive work up revealed FVIII activity < 1% of normal, FVIII inhibitor 13.4 Bethesda Units (BU) per ml, as well as an aPTT of 97 sec without correction by normal plasma. All other coagulation studies were within normal limits. After the diagnosis of acquired hemophilia A (AHA), Factor VIII anti-inhibitor coagulant complex FEIBA® 75 U/kg (Baxter Healthcare Corporation, Westlake Village, CA) was administered every twelve hours as well as methylprednisolone 1 mg/kg once daily. She did not have any other bleeding episodes, but her factor VIII inhibitor levels continued to rise two weeks after initiation of FEIBA® and corticosteroid. Therefore, FEIBA® was discontinued safely, cyclophosphamide (2 mg/kg/day) followed by rituximab (375 mg/m^2^) was added to her regimen until factor VIII inhibitor levels started to decline seven days after initiation of rituximab. She had no further bleeding episodes and her hospitalization course was otherwise uneventful.

## 3. Conclusions

Acquired hemophilia A is a rare bleeding disorder caused by autoantibodies (mostly IgG) directed against clotting FVIII and associated with increased morbidity and mortality. The annual incidence of AHA has been estimated at 0.2–1.0 case per 1 million persons, but this may be underestimated because of difficulty in making the diagnosis (1). The clinical picture is severe hemorrhage in most patients, with a mortality rate up to 22 %. It has been reported that higher mortality risk is associated with higher antibody titers (2, 3). AHA may be associated with underlying malignancies, the postpartum period, drug administration, or autoimmune diseases such as rheumatoid arthritis or systemic lupus erythematosus. There are also case reports of drug-induced hemophilia associated with penicillin, ampicillin, trimethoprim/sulfamethoxazole, clopidogrel, methyldopa, phenytoin, and phenothiazine derivatives (4). No underlying etiology is found in nearly 50% of all cases (2, 5). Patients with AHA tend to present with hemorrhage into the skin, mucous membranes, muscles, soft tissues, or as postpartum in contrast to congenital hemophiliacs in whom hemarthrosis is a typical presentation (6, 7). 

The diagnosis of AHA can be very difficult, as there is rarely a personal or familial history of abnormal bleeding. Laboratory tests reveal prolonged aPTT whereas prothrombin and bleeding times are within normal limits. A mixing study of 1:1 patient-to-control plasma shows an uncorrectable aPTT. Direct measurements of the various factors and inhibitors should be considered next (8). Bleeding control in an acute setting can be achieved by two options: FVIII inhibitor bypassing agents or raising the level of circulating FVIII and elimination of the FVIII inhibitors (7).

### 3.1. FVIII Inhibitor Bypassing Agents

Both recombinant activated factor VII (rFVIIa) and the activated Prothrombin complex concentrate (aPCC) factor 8 inhibitor bypassing activity (FEIBA®) is proven to be effective first line treatment in AHA (5, 9, 10). Goudemand et al. reviewed the use of FEIBA® for the treatment of patients with AHA. FEIBA® was administered at a median dosage of 68 U/kg every 8 to 24 hours for a median of 3.5 days and was found to provide an excellent or good hemostatic efficacy in 89% of the cases (11). Another study by Sallah et al. reported a complete response in 76% of severe AHA cases and 100% of moderate bleeding episodes, for an overall complete response rate of 86% with an average dose of 75 U/kg every 8 to 12 hours (12). Hay et al. studied efficacy of recombinant FVIIa in a multicenter retrospective analysis. The average starting dose was 90mcg/kg every 2 to 6 hours; while a median of 28 doses were given per episode, over a median 3.9 days. The authors reported clinical response in 100% of patients when rFVIIa was administered as a first-line treatment, and in 75% of patients if used as salvage therapy (13). Additionally, rFVIIa might be preferred over aPCC by some clinicians because of its viral safety profile as a recombinant product although there is no comparative study between aPCC and rFVII in terms of efficacy and risk of adverse events (8).

### 3.2. Immunosuppressive Therapy

With this approach, the elimination of the cell line responsible for the production of the antibodies is the goal. In a randomized prospective study by Green et al. AHA patients were treated with prednisone 1 mg/kg/day for 3 weeks. If the autoantibody was still detectable after that treatment, the patients were randomized to receive six weeks either prednisone alone, prednisone with oral cyclophosphamide (2 mg/kg/day), or cyclophosphamide alone. Green et al. reported clinical response to prednisone only in one-third of the patients, whereas 50% of the steroid resistant patients responded to cyclophosphamide- containing regimens (14).

### 3.3. Other Therapies

Rituximab (a monoclonal antibody against B-cell antigen CD20) has been successfully used in treatment of lymphoproliferative and autoimmune disorders. Wiestner et al. reported successful treatment of three patients with high-titer FVIII antibodies who received up to four weekly infusions of rituximab, 375 mg/m2 and demonstrated rapid clinical improvement. Another study by Stasi et al. included 10 patients, eight patients achieved complete remission and two did not respond to rituximab (15, 16). Even though, rituximab has been reported to be an effective treatment of AHA in most case reports and small case series, it should be considered as second-line therapy in patients who fail to respond to first-line treatment. Our patient did not respond to glucocorticoid and cyclophosphamide, and her antibody titer persisted in rising even after that treatment. Her antibody titer demonstrated significant decrease only after addition of rituximab, and remained very low at two, four, and eight weeks.

This report presents a rare bleeding disorder which mandated surgical interventions, and initial hemostasis was quite challenging. Bleeding was stopped after proper diagnosis and primary treatment, but the causative agent (FVIII antibodies) was not eradicated until rituximab was started. This case reemphasizes that the diagnosis of AHA should be considered in any patient with unexplained bleeding, prolonged aPTT, and presence of an autoimmune disease. It highlights the importance of a multidisciplinary approach for appropriate treatment and prevention of recurrence of such a rare though life-threatening condition.
